# Clinical characteristics and pathogen spectrum of patients with fungal rhinosinusitis and risk factors for invasiveness in Northern China: a retrospective study (2015–2024)

**DOI:** 10.1186/s12879-026-14116-6

**Published:** 2026-07-29

**Authors:** Na Li, Mengran Yang, Xiangyi Liu, Xiaobo Tian

**Affiliations:** Department of Laboratory Medicine, Beijing Tongren Hospital, Capital Medical University, Beijing, 100176 China

**Keywords:** Fungal rhinosinusitis, Invasive fungal rhinosinusitis, *Aspergillus*, Diabetes mellitus, Risk factors

## Abstract

**Background:**

Fungal rhinosinusitis (FRS) is becoming increasingly common but remains underdiagnosed or misdiagnosed due to atypical symptoms in invasive cases and limited regional epidemiological data. The aim of this study was to systematically describe the pathogenic spectrum, clinical characteristics, and risk factors for invasive disease in patients with FRS in Northern China over the past decade.

**Methods:**

This retrospective study included 511 patients diagnosed with FRS at a tertiary hospital in Northern China from January 2015 to December 2024. All cases were confirmed by histopathology and fungal culture. Patients were classified as invasive fungal rhinosinusitis (IFRS) or non-invasive fungal rhinosinusitis (NIFRS) based on histopathological evidence of tissue invasion. Demographic characteristics, clinical manifestations, underlying diseases, laboratory findings, and pathogen data were collected and analyzed. Multivariate logistic regression was used to identify independent risk factors for IFRS.

**Results:**

Among 511 patients, 52 (10.2%) were classified as IFRS and 459 (89.8%) as NIFRS. Chronic infection predominated (93.3%), with the maxillary sinus being the most frequently involved site (86.7%). *Aspergillus* species were the predominant pathogens (64.6%), primarily *Aspergillus fumigatus* (25.8%) and *Aspergillus flavus* (25.6%). Bacterial co-infection was present in 47.2% of patients. Compared to NIFRS, IFRS patients had significantly higher frequencies of ocular symptoms (50.0% vs. 16.1%, *P* < 0.001) and nasofacial swelling (34.6% vs. 14.6%, *P* < 0.001), but lower frequencies of nasal obstruction and rhinorrhoea (44.2% vs. 80.2%, *P* < 0.001). The detection rate of Mucorales was significantly higher in IFRS (9.6% vs. 0.9%, *P* < 0.001). Multivariate analysis showed that diabetes mellitus was independently associated with IFRS (OR = 5.187, 95% *CI*: 2.576–10.446, *P* < 0.001). Subgroup analysis revealed distinct clinical and pathological profiles between acute and chronic IFRS.

**Conclusions:**

FRS in Northern China is predominantly chronic and Aspergillus-driven. Ocular symptoms and nasofacial swelling are critical warning signs of invasive disease, and diabetes mellitus was independently associated with IFRS. Clinicians should maintain a high index of suspicion for IFRS in diabetic patients presenting with atypical symptoms to enable early diagnosis and timely intervention.

## Clinical trial number

Not applicable.

## Introduction

Fungal rhinosinusitis (FRS) is an inflammatory condition resulting from fungal infection or invasion of the nasal and paranasal sinus mucosa [1, 2]. Its pathogenesis is closely linked to fungal virulence, host immune status, and perturbations of the local microenvironment. The clinical incidence of FRS has increased in recent years [3–5], a trend largely attributed to the widespread use of broad-spectrum antibiotics and glucocorticoids, the expanding immunocompromised population, and advances in diagnostic modalities [6]. Consequently, FRS has emerged as a common entity in otolaryngology–head and neck surgery.

Based on the extent of tissue invasion, FRS is categorised into invasive fungal rhinosinusitis (IFRS) and non-invasive fungal rhinosinusitis (NIFRS) [1, 2, 7]. IFRS is characterised by rapid progression, a propensity to invade adjacent structures, including the orbit, cranial cavity, and vasculature, and severe clinical manifestations with attendant complications. It is associated with high morbidity and mortality and constitutes an otolaryngological emergency [8–11].

The aetiological agents of FRS are diverse, with *Aspergillus* spp., Mucorales, *Candida* spp., and other genera frequently implicated [12, 13]. Aetiological profiles exhibit geographical variation attributable to differences in climate, residential environment, and other regional factors [14, 15]. Moreover, clinical phenotypes are closely related to the causative fungal species, underlying diseases, and host immune status [10, 16, 17].

To date, large-scale systematic investigations of FRS in Northern China remain limited. The aetiological spectrum, clinical characteristics, and risk factors for disease progression have not been fully characterised, hindering early diagnosis and precision treatment. Moreover, findings from international studies are not directly applicable to this region due to geographical and ethnic disparities, and most existing studies are small retrospective analyses.

To address this critical research gap, we conducted a 10-year retrospective analysis of 511 FRS patients admitted to a tertiary medical center in Northern China from 2015 to 2024, aiming to delineate the regional pathogenic spectrum, characterize the distinct clinical features of IFRS, and identify factors associated with the invasive phenotype of FRS.

## Materials and methods

### Study population

Complete clinical data were retrospectively collected from 511 patients diagnosed with FRS who were presented to and treated at Beijing Tongren Hospital, Capital Medical University, between January 2015 and December 2024.

Inclusion criteria were as follows: (1) definitive diagnosis of FRS confirmed by clinical manifestations (nasal obstruction, nasal discharge, nasofacial swelling, visual impairment, etc.), imaging findings (sinus cavities filled with heterogeneous soft tissue density shadows, calcified spots within the lesion area, bone destruction, etc.), nasal endoscopy results (yellow, brown, or blackish-brown caseous masses at the sinus ostium; diffuse mucosal hyperaemia, erosion, or ulcers accompanied by greyish-black necrotic tissue, etc.), etiologic testing (successful fungal isolation), and histopathological examination (fungal invasion of nasal mucosa, sinus tissues, or bone) [13]; (2) complete and traceable clinical data, including demographic characteristics, symptoms and signs, imaging findings, past medical history, and laboratory test results.

Exclusion criteria were: (1) concomitant severe systemic infectious diseases (e.g., sepsis, severe pneumonia) that might interfere with the study outcomes; (2) incomplete clinical data with missing key variables precluding statistical analysis; (3) use of glucocorticoids or antifungal agents within one week before enrollment, which could affect disease assessment and etiologic detection.

A total of 655 patients with suspected FRS were initially screened from the database. After review, 144 cases were excluded: 61 due to missing fungal culture results (40 were histopathology positive but culture negative, and 21 did not undergo fungal culture); 47 due to lack of histopathological data (mostly nasal swab specimens that were culture positive but either histopathology negative or not examined); 8 had incomplete clinical data and 4 had severe pneumonia; and 24 due to use of glucocorticoids or antifungal agents within one week before enrollment.

Thus, the study cohort consisted of patients with both histopathological confirmation and positive fungal culture, and our findings on pathogen distribution apply primarily to culture-confirmed FRS.

### Data collection

Clinical data were retrieved from the Hospital Information System (HIS), Laboratory Information System (LIS), and Jiahe company Medical Record System. The following data were extracted:

1. **Demographic data**: sex, age, and place of residence;
2. **Clinical symptoms and signs**: nasal obstruction, rhinorrhoea, headache, ocular symptoms (decreased visual acuity, blindness, diplopia, orbital cellulitis, orbital apex syndrome, proptosis, etc.), nasofacial swelling, oral symptoms (toothache, gingival swelling), and ear symptoms (tinnitus, hearing loss);
3. **Past medical history**: hypertension, diabetes mellitus, malignant tumours, smoking history, history of hormone use, nasal septum deviation, and nasal polyps. Diabetes mellitus was defined as a documented diagnosis in the medical history, or HbA1c ≥ 6.5%, or fasting blood glucose ≥ 7.0 mmol/L;
4. **Imaging findings**: sinus inflammation and extent of involvement as revealed by sinus computed tomography (CT) or magnetic resonance imaging (MRI);
5. **Laboratory and microbiological data**: results of fungal and bacterial cultures and identification; white blood cell (WBC), neutrophil (NEUT), and eosinophil (EOS) counts measured by haematology analyser; blood glucose levels measured by biochemistry analyser.

### Pathogen diagnosis

Specimens were collected by clinicians following standard protocols. Nasal swabs were inoculated onto Sabouraud dextrose agar (SDA) via three-zone streaking, while fungal clumps or sinus tissue specimens were sectioned into pieces and spot-inoculated onto SDA. Plates were incubated at 27 °C for 2 to 14 days for fungal culture. The same specimens were simultaneously inoculated onto blood agar and chocolate agar via three-zone streaking and incubated at 35 °C in 5% CO₂ for 18 to 24 h for aerobic bacteria, and onto anaerobic blood agar at 35 °C in an anaerobic chamber for 48 to 72 h for anaerobic bacteria.

Pathogens were identified by combining colony morphology, microscopic characteristics (Gram staining, lactophenol cotton blue staining and fluorescent staining), and matrix-assisted laser desorption/ionization time-of-flight mass spectrometry (MALDI-TOF MS). For yeast-like fungi, the in-situ formic acid extraction method was employed; for filamentous fungi, the double formic acid method was used; for bacteria, the direct target plate spotting method was adopted. One microliter of matrix solution (α-cyano-4-hydroxycinnamic acid, CHCA) was added, and the target plate was air-dried at room temperature before being placed into the mass spectrometer for detection. A MALDI-TOF MS score > 2.0 indicated reliable species identification, while a score of 1.7–2.0 indicated reliable genus identification [18, 19].

### Pathological diagnosis

Sinus tissue specimens were fixed in formaldehyde, embedded in paraffin, sectioned, and stained with haematoxylin and eosin (H.E). The presence of fungal invasion, tissue necrosis, and inflammatory cell infiltration was examined microscopically.

Diagnostic criteria for IFRS were defined as the presence of fungal hyphae within tissues accompanied by tissue invasion and necrosis. NIFRS was diagnosed when fungi colonised only the sinus mucosal surface or formed fungal balls without evidence of tissue invasion [13].

### Statistical analysis

Statistical analyses were performed using SPSS version 27.0.1. Categorical variables were expressed as counts (percentages) and compared between groups using the chi-square test or Fisher’s exact test, as appropriate. Continuous variables were first assessed for normality using the Kolmogorov–Smirnov test. Normally distributed data were expressed as mean ± standard deviation (x̄ ± s) and compared using the independent samples t-test; non-normally distributed data were expressed as median with interquartile range [*M (Q1*,* Q3)*] and compared using the Mann–Whitney U test.

Multivariate logistic regression analysis was performed to identify independent risk factors for IFRS. Variables with *P* < 0.05 in univariate analysis were entered into the regression model, together with clinically relevant variables based on prior literature (age, sex, hypertension, diabetes mellitus, and malignant tumors). Given the limited number of IFRS events (*n* = 52), to minimize overfitting, the final model retained five predictors, including four variables with statistically significant differences and neutrophil count as a marker of systemic inflammation, yielding an events per variable (EPV) ratio of approximately 10.4 to ensure model stability. Model calibration was assessed using the Hosmer-Lemeshow goodness-of-fit test. Multicollinearity was assessed using variance inflation factors (VIF). Results were reported as odds ratios (OR) with 95% confidence intervals (CI). Graphs were generated using GraphPad Prism version 8.3.0. A two-sided *P* value < 0.05 was considered statistically significant.

### Ethics

This retrospective study was conducted using anonymised data from the patient cohort after approval by the Ethics Committee of Beijing Tongren Hospital, Capital Medical University (Approval No. TREC2024-KYS244). Written informed consent was waived due to the retrospective nature of the study, in accordance with national legislation and institutional requirements.

## Results

### Characteristics of the study population

**Fig. 1:**
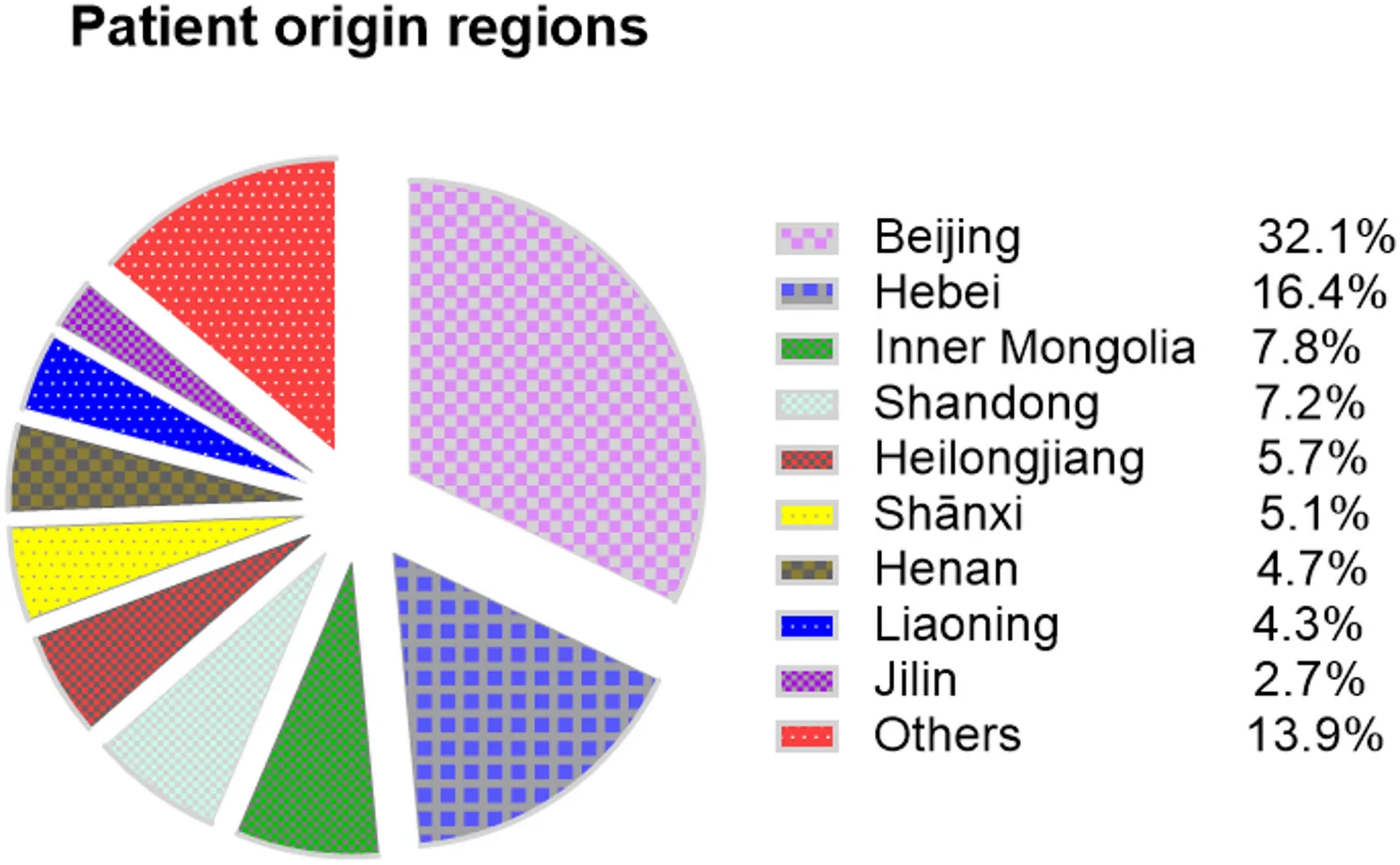
Residential distribution of 511 patients with fungal rhinosinusitis

Among the 511 patients with FRS, 231 were male (45.2%) and 280 were female (54.8%). The patients were predominantly from Beijing (32.1%), Hebei (16.4%), Inner Mongolia (7.8%), and Shandong (7.2%), encompassing multiple provinces in Northern China (Fig. 1).

Chronic infection comprised 93.3% (477/511) of cases, whereas acute infection accounted for 6.7% (34/511), with a statistically significant difference (χ²=768.098, *P* < 0.001). The IFRS group constituted 10.2% (52/511) of the cohort, including 34 cases of acute infection and 18 cases of chronic infection; the NIFRS group comprised 89.8% (459/511), with a statistically significant difference between groups (χ² = 648.333, *P* < 0.001). Patients in the IFRS group were significantly older (57.9 ± 15.9 years) than those in the NIFRS group [53.0 (40.0, 62.0) years] (Z=–2.946, *P* = 0.003).

Among the 459 patients with NIFRS, 224 (48.8%) were classified as fungal ball and 54 (11.8%) as allergic fungal rhinosinusitis (AFRS). The remaining 181 patients (39.4%) lacked sufficient documentation for reliable subtyping.

The maxillary sinus was the most frequently involved site, affected in 443 cases (86.7%, 443/511), including 124 cases of single sinus involvement, 232 cases of multiple sinus involvement, and 87 cases of total sinus involvement. The ethmoid sinus was the second most commonly involved site, whereas the sphenoid and frontal sinuses exhibited relatively lower involvement rates (Table 1).

**Table 1:** Characteristics of sinus involvement in FRS

Sinus Type	Single Sinus (*n*)	Multiple Sinus (*n*)	Total Sinus (*n*)	Total (*n*)	Proportion (%, *n* = 511)
Maxillary Sinus	124	232	87	443	86.7
Ethmoid Sinus	31	188	87	306	59.9
Sphenoid Sinus	27	131	87	245	47.9
Frontal Sinus	8	65	87	160	31.3

Note: Multiple sinus involvement refers to involvement of 2 or 3 sinuses, Total sinus involvement refers to involvement of all 4 sinuses, FRS: fungal rhinosinusitis

### Fungal pathogen spectrum and mixed infection

A total of 520 pathogenic fungal strains were isolated, comprising 336 strains of *Aspergillus* (64.6%), 152 strains of non-*Aspergillus* filamentous fungi (29.2%), and 32 strains of yeasts (6.2%). *Aspergillus fumigatus* (134 strains, 25.8%) and *Aspergillus flavus* (133 strains, 25.6%) were the predominant pathogens; among non-*Aspergillus* fungi, *Alternaria alternata* (32 strains, 6.2%) and *Schizophyllum commune* (30 strains, 5.8%) were the most frequently isolated. Mucorales fungi were detected in five patients in the IFRS group (9.6%, 5/52) and four patients in the NIFRS group (0.9%, 4/459), with a statistically significant difference between the two groups (χ² = 15.895, *P* < 0.001) (Table 2).

**Table 2:** Classification and proportion of pathogenic fungi in 511 patients with FRS

Fungal classification	Fungal species	Number of strains (*n* = 520)	Proportion (%)
*Aspergillus*	*Aspergillus fumigatus*	134	25.8
	*Aspergillus flavus*	133	25.6
	*Aspergillus versicolor*	20	3.9
	*Aspergillus niger*	14	2.7
	*Aspergillus oryzae*	14	2.7
	*Aspergillus terreus*	11	2.1
	*Aspergillus nidulans*	6	1.2
	*Aspergillus lentulus*	3	0.6
	*Aspergillus japonicus*	1	0.2
Other filamentous fungi	*Alternaria alternata*	32	6.2
	*Schizophyllum commune*	30	5.8
	*Penicillium* spp.	21	4.1
	*Exophiala* spp.	12	2.3
	*Fusarium* spp.	13	2.5
	*Scedosporium* spp.	11	2.1
	*Rhizopus* and *Rhizomucor* spp.	9	1.7
	*Cladosporium* spp.	9	1.7
	*Curvularia* spp.	3	0.6
	*Acremonium* spp.	3	0.6
	Others	9	1.7
Yeast-like fungi	*Candida* spp.	28	5.4
	*Cryptococcus* spp.	2	0.4
	*Malassezia* spp.	2	0.4

Note: The Mucorales isolates comprised predominantly *Rhizopus* spp. (*n* = 7) and *Rhizomucor* spp. (*n* = 2), FRS: fungal rhinosinusitis

Bacterial co-infection was observed in 241 patients (47.2%), including 219 patients with single bacterial infection and 22 patients with two or more bacterial species. A total of 286 bacterial strains were isolated, comprising 249 aerobic strains (87.1%) and 37 anaerobic strains (12.9%). The predominant aerobic bacteria were *Staphylococcus aureus* (58 strains, 20.3%), *Klebsiella* spp. (33 strains, 11.5%), and *Streptococcus* spp. (25 strains, 8.7%). The main anaerobic isolates were *Propionibacterium spp*. (23 strains, 8.0%) and *Prevotella spp*. (6 strains, 2.1%) (Table 3).

**Table 3:** Classification and proportion of concurrent bacteria in 511 FRS patients

Bacterial category	Bacterial species	Number of strains (*n* = 286)	Proportion (%)
Aerobic bacteria	*Staphylococcus aureus*	58	20.3
	*Klebsiella* spp.	33	11.5
	*Streptococcus* spp.	25	8.7
	*Pseudomonas* spp.	23	8.0
	*Haemophilus* spp.	20	7.0
	*Enterobacter* spp.	16	5.6
	*Citrobacter* spp.	15	5.2
	*Stenotrophomonas* spp.	10	3.5
	*Escherichia* spp.	7	2.4
	*Staphylococcus lugdunensis*	6	2.1
	*Serratia marcescens*	6	2.1
	Other Aerobic Bacteria	30	10.5
Anaerobic bacteria	*Propionibacterium* spp.	23	8.0
	*Prevotella* spp.	6	2.1
	Other Anaerobic Bacteria	8	2.8

Note: FRS: fungal rhinosinusitis

### Differences in clinical manifestations between IFRS and NIFRS

The incidences of ocular symptoms (50.0% vs. 16.1%) and nasofacial swelling (34.6% vs. 14.6%) were significantly higher in the IFRS group than in the NIFRS group, whereas the incidences of nasal obstruction and rhinorrhoea (44.2% vs. 80.2%) were significantly lower (all *P* < 0.001). No significant differences were observed between the two groups regarding the incidences of headache, oral symptoms, or ear symptoms (*P* > 0.05) (Table 4).

**Table 4:** Differences in symptoms between IFRS group and NIFRS group (*n*/%)

Symptoms/Signs	IFRS group (*n* = 52)	NIFRS group (*n* = 459)	χ2	*P*
Ocular symptoms	26(50.0)	74(16.1)	34.059	< 0.001
Nasofacial swelling	18(34.6)	67(14.6)	13.498	< 0.001
Headache	27(51.9)	227(49.5)	0.114	0.736
Oral symptoms	9(17.3)	42(9.2)	3.459	0.063
Ear symptoms	2(3.8)	18(3.9)	0.123	0.726
Nasal obstruction and nasal discharge	23(44.2)	368(80.2)	33.583	< 0.001

Note: IFRS: invasive fungal rhinosinusitis, NIFRS༚non-invasive fungal rhinosinusitis

Within the IFRS group, subgroup comparison between acute invasive fungal rhinosinusitis (AIFRS) and chronic invasive fungal rhinosinusitis (CIFRS) revealed that ocular symptoms were more frequent in AIFRS [20/34 (58.8%) vs. 6/18 (33.3%)] (*P* = 0.080), while nasofacial swelling was more common in CIFRS [8/18 (44.4%) vs. 10/34 (29.4%)] (*P* = 0.278), and headache was also more frequent in CIFRS [11/18 (61.1%) vs. 16/34 (47.1%)] (*P* = 0.335), however, none of these differences reached statistical significance.

### Univariate and multivariate analysis

No significant differences were observed between the IFRS group (*n* = 52) and the NIFRS group (*n* = 459) regarding gender, smoking history, nasal septum deviation, nasal polyps, or history of hormone use (all *P* > 0.05). However, significant differences were found between the two groups in age, hypertension, diabetes mellitus, history of malignant tumours, white blood cell (WBC) count, neutrophil (NEUT) count, and eosinophil (EOS) count (all *P* < 0.05). Patients in the IFRS group were older and had higher incidences of hypertension, diabetes mellitus, and history of malignant tumours compared with those in the NIFRS group (Table 5).

**Table 5:** Univariate analysis of risk factors for IFRS(*n*/%, *x̄±s*,* M (Q1*,* Q3)*)

Observation indicators	IFRS group (*n* = 52)	NIFRS group (*n* = 459)	χ2 / Z	*P*
Gender			1.744	0.187
Male	28(53.8)	203(44.2)		
Female	24(46.2)	256(55.8)		
Age	57.9 ± 15.9	53.0(40.0, 62.0)	-2.946	0.003
Smoking History (including ex-smokers)	10(19.2)	64(13.9)	1.054	0.304
Hypertension	21(40.4)	95(20.7)	10.317	0.001
Diabetes Mellitus	25(48.1)	55(12.0)	46.084	< 0.001
Nasal Septum Deviation	11(21.1)	69(15.0)	1.325	0.249
Nasal Polyps	5(9.6)	64(13.9)	0.424	0.515
Malignant Tumors	11(21.2)	41(8.9)	7.632	0.006
Hormone Use History	7(13.5)	49(10.7)	0.372	0.542
WBC (×10⁹/L)	7.72(5.16,11.58)	5.82(4.82,6.89)	-4.199	< 0.001
NEUT (×10⁹/L)	5.21(3.29, 8.89)	3.30(2.55, 4.19)	-5.086	< 0.001
EOS (×10⁹/L)	0.05(0.01,0.11)	0.12(0.06,0.21)	-5.220	< 0.001

Note: Malignant tumors mainly include non-Hodgkin lymphoma (nasal type), leukemia, nasopharyngeal carcinoma, and malignant tumors in other sites, IFRS: invasive fungal rhinosinusitis, NIFRS: non-invasive fungal rhinosinusitis, WBC: white blood cell, NEUT: neutrophil, EOS: eosinophil

Further subgroup analysis within IFRS showed that the prevalence of diabetes mellitus was higher in the AIFRS group than in the CIFRS group [18/34 (52.9%) vs. 7/18 (38.9%), *P* = 0.334]. CIFRS patients had a higher proportion of malignant tumors [4/18 (22.2%) vs. 7/34 (20.6%), *P* = 0.826], and Mucorales infection was identified exclusively in the AIFRS subgroup [5/34 (14.7%) vs. 0/18, *P* = 0.224]. However, none of these differences reached statistical significance.

Multivariate logistic regression analysis was performed with IFRS as the dependent variable (yes = 1, no = 0). The independent variables included age, hypertension, diabetes mellitus, malignant tumours, and NEUT count, among which hypertension, diabetes mellitus, and malignant tumours were categorical variables. Diabetes mellitus was independently associated with IFRS (OR = 5.187, 95% *CI*: 2.576–10.446, *P* < 0.001) (Table 6).

**Table 6:** Multivariate logistic regression analysis of risk factors for IFRS

Variables	B	SE	Waldχ^2^	Sig.	OR	95% CI	
Age	0.011	0.013	0.732	0.392	1.011	0.986	1.036
Hypertension	-0.302	0.390	0.599	0.439	0.74	0.344	1.588
Diabetes mellitus	1.646	0.357	21.242	< 0.001	5.187	2.576	10.446
Malignant tumors	-0.751	0.427	3.091	0.079	0.472	0.204	1.090
NEUT (×10⁹/L)	0.297	0.056	27.619	< 0.001	1.346	1.205	1.503

Note: B: Regression coefficient, SE: Standard error, Wald χ²: Wald chi-square value, Sig.: Significance level, OR: Odds ratio, CI: Confidence interval, IFRS: invasive fungal rhinosinusitis, Malignant tumors mainly include non-Hodgkin lymphoma (nasal type), leukemia, nasopharyngeal carcinoma, and malignant tumors in other sites, NEUT: neutrophil

The Hosmer-Lemeshow test indicated acceptable model calibration (χ² =11.706, df = 8, *P* = 0.165). Collinearity diagnostics for the final model showed that all VIF values were below 1.3 (VIF: age 1.270, hypertension 1.227, diabetes mellitus 1.112, malignant tumors 1.015, NEUT 1.036), indicating no substantial multicollinearity.

**Fig. 2:**
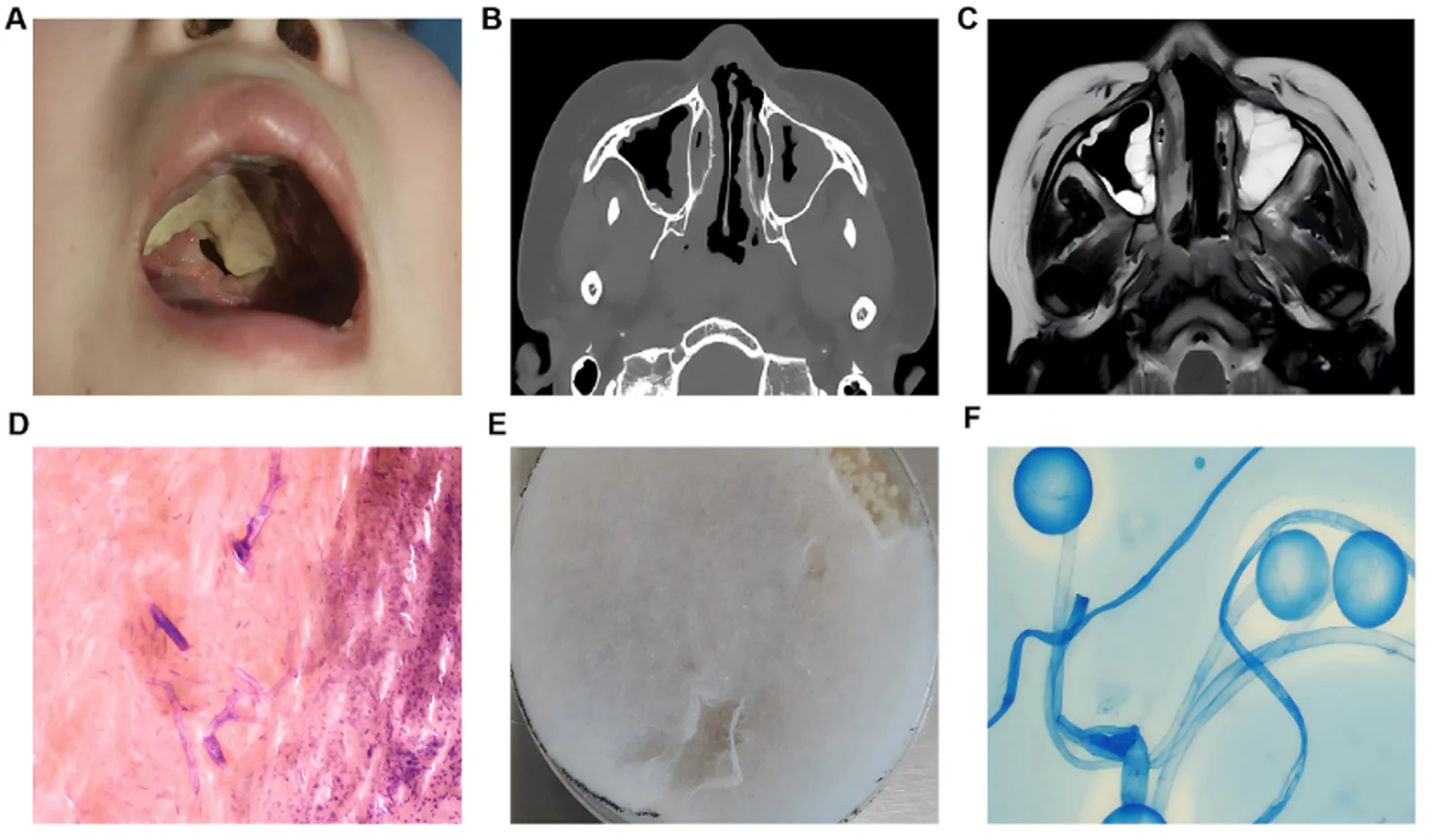
Acute invasive fungal rhinosinusitis caused by *Rhizopus* (Case 1). (**A**) Black-brown dry crust on the hard palate. (**B**) CT scan showing sinusitis. (**C**) MRI showing extensive invasion. (**D**) Histopathology showing hyphae invading cartilage (haematoxylin and eosin stain). (**E**) Rhizopus culture on SDA. (**F**) Microscopic examination of *Rhizopus* (lactophenol cotton blue stain). The patient had type 1 diabetes mellitus and diabetic ketoacidosis

**Fig. 3:**
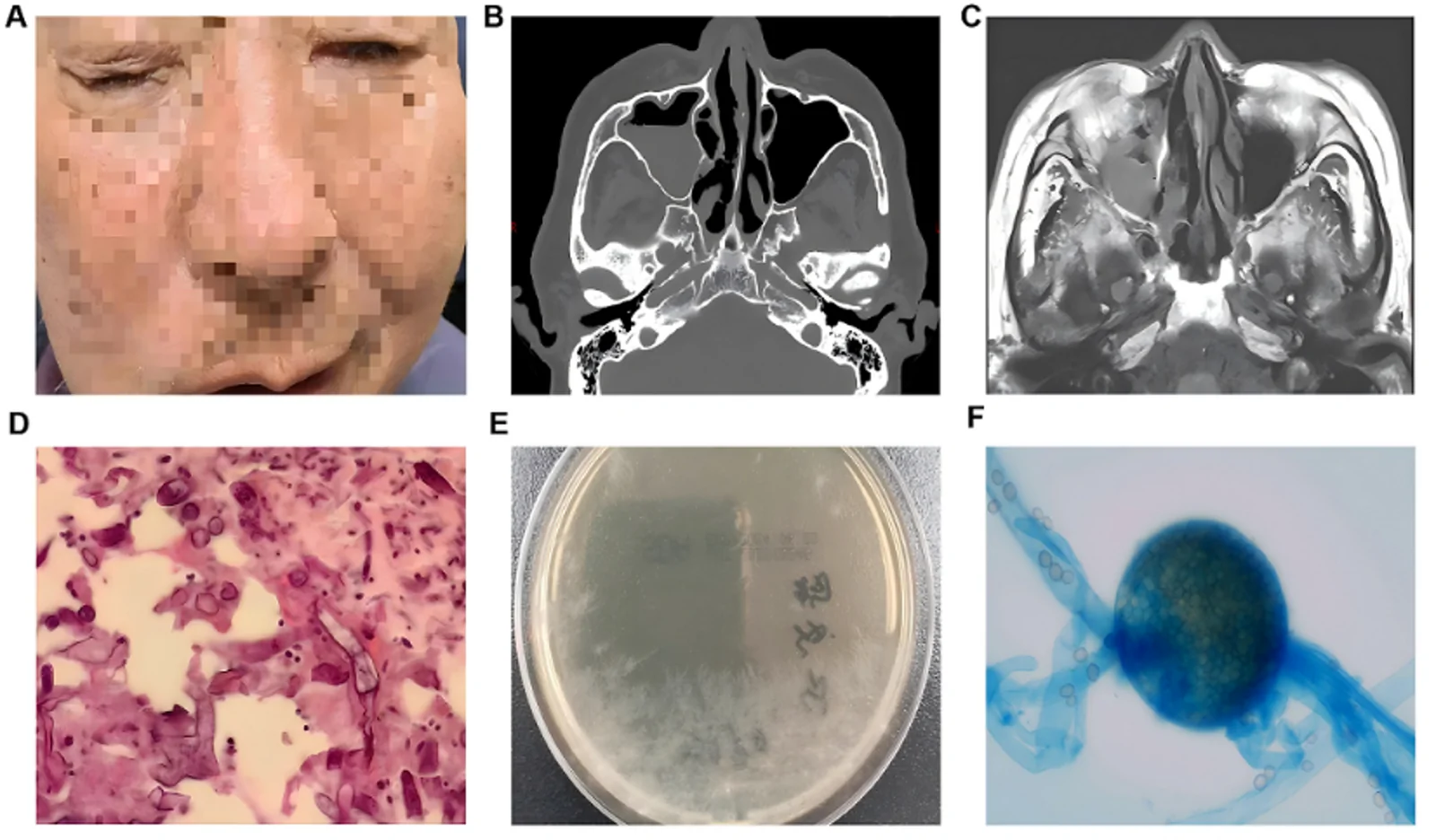
Acute invasive fungal rhinosinusitis caused by *Rhizopus* (Case 2). (**A**) Right facial swelling accompanied by ptosis. (**B**) CT scan showing bilateral ethmoiditis and right maxillary sinusitis. (**C**) MRI showing lesions involving the cavernous sinus and pterygopalatine fossa. (**D**) Histopathology showing fungal invasion of surrounding tissues (haematoxylin and eosin stain). (**E**) *Rhizopus* culture on SDA. (**F**) Microscopic examination of *Rhizopus* (lactophenol cotton blue stain). The patient had a history of diabetes mellitus (poor glycaemic control) and hypertension

**Fig. 4:**
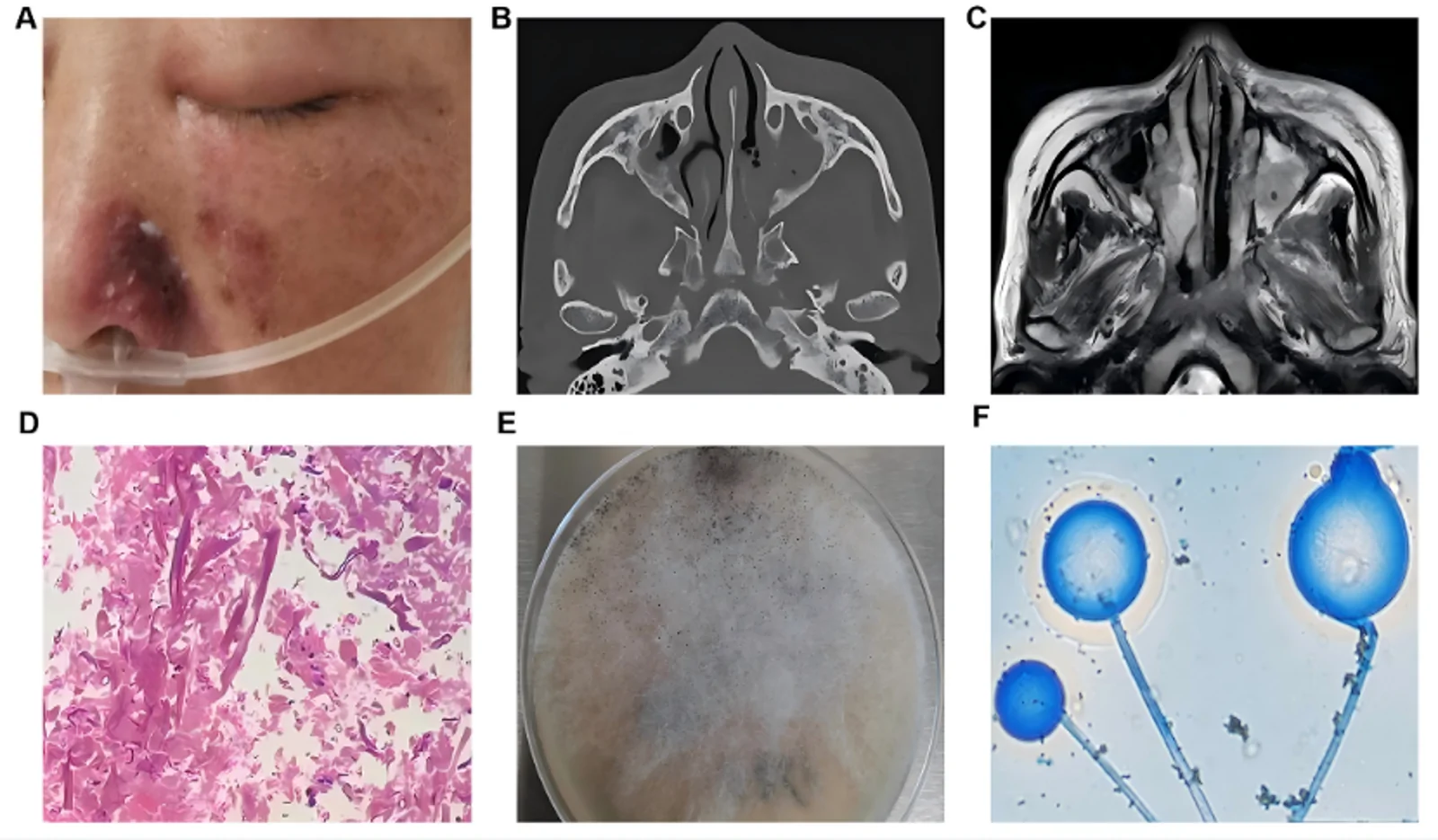
Invasive fungal rhinosinusitis caused by *Rhizopus* (Case 3). (**A**) Mild left facial swelling accompanied by left eye vision loss. (**B**) CT scan showing pansinusitis and nasal septum deviation. (**C**) MRI showing lesions involving the left orbit and multiple groups of sinuses. (**D**) Histopathology showing a large number of fungal hyphae in the tissue (haematoxylin and eosin stain). (**E**) *Rhizopus* culture on SDA. (**F**) Microscopic examination of *Rhizopus* (lactophenol cotton blue stain). The patient had hypertension and a history of type 2 diabetes mellitus with long-term poor glycaemic control

**Fig. 5:**
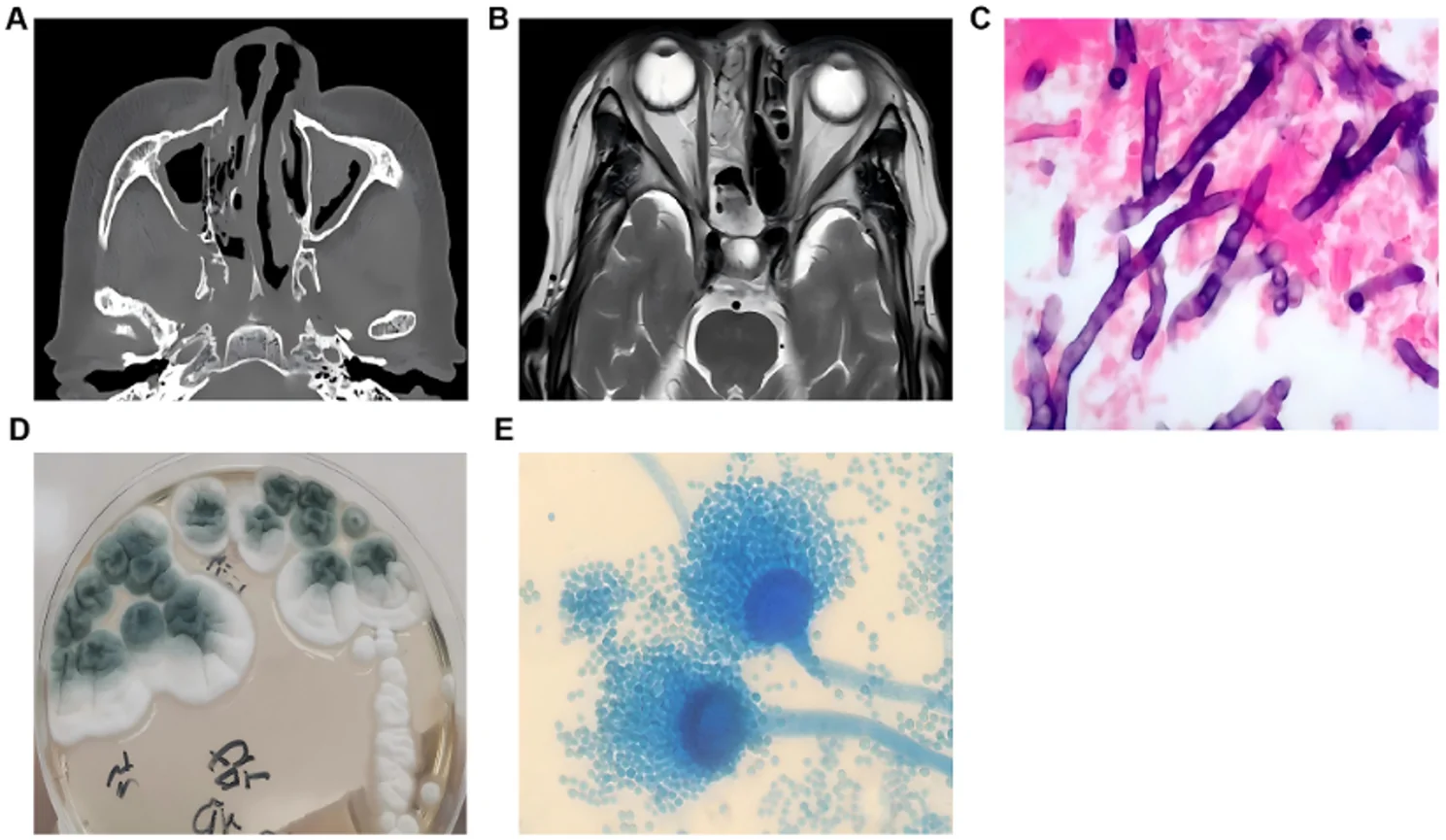
Invasive fungal rhinosinusitis caused by *Aspergillus fumigatus* (Case 4). (**A**) CT scan showing right frontal sinusitis, ethmoiditis, sphenoiditis, and bilateral maxillary sinusitis. (**B**) MRI showing lesions extensively involving the nasal-cranial-orbital structures and multiple groups of sinuses. (**C**) Histopathology showing a large number of *Aspergillus* hyphae in the tissue (haematoxylin and eosin stain). (**D**) *Aspergillus fumigatus* culture on SDA. (**E**) Microscopic examination of *Aspergillus fumigatus* (lactophenol cotton blue stain). The patient had a 10-year history of hypertension and newly diagnosed diabetes mellitus

Patients with IFRS typically presented with pronounced clinical symptoms or signs, frequently accompanied by diabetes mellitus, and exhibited extensive involvement of extrasinus tissues. This invasive pattern was particularly pronounced in cases of Mucorales infection. Similarly, Aspergillus infection in patients with diabetes mellitus could also lead to severe IFRS (Figs. 2, 3, 4, and 5).

### Treatment and medication

Surgical intervention consisting of sinusotomy and debridement of diseased tissue was performed in 477 patients (93.3%, 477/511). Of these, 51.7% (264/511) received topical intranasal corticosteroids, and 7.8% (40/511) received systemic corticosteroids.

Systemic antifungal therapy was administered to 4.9% (25/511) of patients. Among these, the proportion of patients receiving antifungal treatment was significantly higher in the IFRS group (32.7%, 17/52) than in the NIFRS group (1.7%, 8/459) (χ²=96.153, *P* < 0.001). Patients in the IFRS group received adjunctive treatment with liposomal amphotericin B, isavuconazole, or voriconazole administered orally or intravenously.

Postoperatively, treatments to promote drainage (including mucolytics and nasal irrigation) and preventive anti-inflammatory therapy were administered.

## Discussion

FRS is an inflammatory disorder of the nasal and paranasal sinus mucosa triggered by fungal infection, with its pathogenesis closely linked to environmental exposures and host immune competence [4, 10, 14, 15]. In this study, we performed a 10-year retrospective analysis of 511 patients diagnosed with FRS in Northern China, systematically delineating the regional pathogen profile, clinical phenotypes, key risk factors, and current therapeutic paradigms. These findings provide critical regional evidence to inform early identification, risk stratification, and precision management of FRS in clinical practice.

Consistent with prior studies [3, 5], FRS in our cohort was predominantly chronic (93.3%) and most frequently involved the maxillary sinus (86.7%). The predilection for the maxillary sinus may be attributed to its unique structural properties: large volume and relatively high ostium predispose to impaired mucociliary clearance and secretory stasis, thereby creating a permissive microenvironment for fungal spore retention, colonisation, and proliferation. Surgical intervention, primarily functional endoscopic sinus surgery (FESS), was performed in 93.3% of patients; for cases complicated by nasal polyposis or significant septal deviation, concurrent polypectomy or septoplasty was performed to alleviate mechanical obstruction and re-establish physiological sinus ventilation and drainage, consistent with current clinical guidelines [20–22].

This study identifies Aspergillus species as the predominant etiological agents of FRS in Northern China (64.6%), with *Aspergillus fumigatus* (25.8%) and *Aspergillus flavus* (25.6%) being the most commonly isolated species. This pathogen distribution is consistent with epidemiological data reported across Asia and globally [13, 23–26], further confirming the universal predominance of *Aspergillus* spp., particularly *Aspergillus fumigatus* and *Aspergillus flavus*, in FRS pathogenesis worldwide. Of notable clinical relevance, the detection rate of Mucorales fungi was significantly higher in the IFRS subgroup (9.6%) compared with the NIFRS subgroup (0.9%). When integrated with multivariate logistic regression analysis, which showed that diabetes mellitus was independently associated with IFRS (OR = 5.187, *P* < 0.001), these findings establish a critical pathogenic link. Hyperglycaemia not only fosters fungal proliferation by providing a nutrient-rich microenvironment but also compromises host innate immunity through complex immunometabolic perturbations, including impairment of neutrophil chemotaxis, migration, and neutrophil extracellular trap (NET) formation, as well as activation of protein kinase C (PKC) signalling pathways—collectively attenuating the host’s capacity to clear invasive fungi [27]. This dysregulated immune microenvironment confers a selective advantage to Mucorales fungi, which are inherently angioinvasive and rapidly proliferative. From a pathophysiological perspective, this explains the heightened predisposition of diabetic IFRS patients to orbital invasion (manifested by a high incidence of ocular symptoms) and rapidly progressive facial soft tissue involvement (presenting as nasofacial swelling), which are associated with severe complications and poor clinical outcomes [28–31]. Consequently, these findings underscore a pivotal clinical principle: for diabetic patients presenting with sinusitis symptoms, especially those accompanied by ocular manifestations (e.g., visual deterioration, diplopia, eyelid oedema) or progressive nasofacial swelling, IFRS, particularly mucormycosis, should be prioritised in the differential diagnosis, and urgent invasive etiological evaluation (including histopathological examination and deep tissue fungal culture) must be initiated [32]. Notably, given that ocular symptoms may be the presenting feature and, in some cases, even the sole manifestation in the absence of prominent sinonasal complaints, affected patients often initially present to ophthalmologists. Therefore, clinicians, particularly ophthalmologists, should maintain a high level of awareness for differential diagnosis to avoid misdiagnosis and delayed treatment.

Beyond the overall comparison between IFRS and NIFRS, subgroup analysis further revealed heterogeneity between AIFRS and CIFRS. Although the differences in diabetes mellitus, malignant tumors, and Mucorales infection did not reach statistical significance, the observed trends still point to distinct underlying mechanisms. CIFRS patients tended to have a higher proportion of malignant tumors, predominantly hematological malignancies, which aligns with the notion that CIFRS often develops in the setting of chronic immunosuppression. In contrast, Mucorales infection was found exclusively in the AIFRS subgroup, consistent with its well-recognized fulminant and angioinvasive behavior. The higher frequency of ocular symptoms in AIFRS further supports its aggressive propensity for orbital involvement. These divergent patterns suggest that the immune defects and clinical trajectories of AIFRS and CIFRS are fundamentally different, which may carry implications for diagnostic workup and treatment planning.

Notably, NIFRS patients exhibited significantly higher peripheral blood EOS counts than IFRS patients (0.12 × 10⁹/L vs. 0.05 × 10⁹/L, *P* < 0.001). This difference likely reflects the distinct immunological profiles of these conditions: elevated EOS in NIFRS may indicate an allergic phenotype, particularly allergic fungal rhinosinusitis (AFRS), contrasting sharply with the immunocompromised state characteristic of IFRS. In our cohort, 224 NIFRS patients were classified as fungal ball and 54 as AFRS, further supporting the heterogeneity of NIFRS and the association between eosinophilia and the allergic subtype. It is important to distinguish true predisposing risk factors from disease-related markers. While univariate analysis showed significant differences in WBC, neutrophil, and eosinophil counts between IFRS and NIFRS groups, these parameters should be interpreted as markers of systemic inflammation or immune dysregulation secondary to the invasive process. In the multivariate analysis, neutrophil count remained independently associated with IFRS (OR = 1.346), however, this association does not reflect a causal relationship and likely represents an inflammatory manifestation of IFRS rather than an independent causal risk factor. In contrast, diabetes mellitus represents a true predisposing factor that precedes and mechanistically contributes to IFRS development.

Additionally, concurrent bacterial infection was observed in 47.2% of FRS patients, with *Staphylococcus aureus* (20.3%) and *Klebsiella* spp. (11.5%) as the predominant bacterial pathogens. The high rate of bacterial co-infection complicates the management of FRS. In IFRS cases requiring aggressive treatment, antifungal monotherapy may therefore be insufficient, and targeted antibacterial therapy should be incorporated based on microbiological findings [33, 34]. The predominance of *Staphylococcus aureus* and *Klebsiella* spp. may reflect a mixture of community-acquired and healthcare-associated pathogens, warranting individualised anti-infective strategies.

IFRS and NIFRS represent distinct extremes of the FRS disease spectrum, with fundamental differences in clinical presentation and therapeutic needs. Our data clearly show that the IFRS group had significantly higher rates of ocular symptoms (50.0%) and nasofacial swelling (34.6%) compared with the NIFRS group, whereas the incidence of typical upper respiratory tract symptoms (e.g., nasal obstruction, rhinorrhoea) was markedly lower. This symptomatic discrepancy directly reflects the invasive nature of IFRS, wherein pathological involvement extends beyond the sinus mucosal surface to invade orbital and facial soft tissues outside the sinus bony boundaries [35]. Accordingly, IFRS warrants an urgent and aggressive surgical approach. Early, extensive debridement is the cornerstone of treatment to effectively reduce fungal burden and relieve mass effect. Systemic antifungal therapy should be considered in high-risk patients, particularly those with acute IFRS, diabetes mellitus, orbitocranial involvement, or Mucorales infection [36, 37]. Before initiating systemic antifungals, a thorough assessment of patient tolerance and potential side effects is warranted. In this cohort, only 32.7% (17/52) of IFRS patients received systemic antifungal therapy. According to clinical guidelines, systemic antifungal therapy is definitively indicated for all forms of IFRS, including CIFRS [20, 38]. The lack of treatment in the remaining 35 patients (67.3%) primarily reflects a limitation of the retrospective cohort, such as incomplete documentation of clinical decisions and unrecorded reasons for not prescribing. The documented reasons included contraindications to antifungals, use of only topical therapy, or delayed diagnosis. In contrast, NIFRS, particularly the fungal ball phenotype, is primarily a localised space-occupying and inflammatory disorder. The mainstay of treatment is complete surgical resection of the lesion. For pharmacotherapy, the majority of FRS patients (51.7%) received intranasal corticosteroids to control postoperative mucosal inflammation and prevent disease recurrence. In contrast, systemic corticosteroids were used cautiously (7.8%) in FRS patients due to their potential to induce immunosuppression, which may exacerbate fungal infection [39–41].

Several limitations of this study should be acknowledged. First, our inclusion criteria required both histopathological confirmation and positive fungal culture. During the study period, 40 histopathologically proven FRS cases were excluded due to negative fungal culture results, accounting for 7.3% (40/551) of all histopathology-proven cases. This selection bias may lead to underrepresentation of slow-growing or non-viable fungi in our pathogen spectrum. Consequently, culture-dependent methods may overestimate the proportion of easily cultivable species such as *Aspergillus*. Nonetheless, the consistent predominance of *Aspergillus* across multiple Asian studies suggests that our main finding remains robust. Therefore, our pathogen spectrum and derived conclusions apply primarily to culture-confirmed FRS. It should be noted that histopathology proven but culture negative cases represent clinically significant infections and that fungal cultures have inherent false negative rates. However, species level identification still requires culture methods. In future studies, the integration of molecular diagnostic tools such as PCR or metagenomic next generation sequencing may help overcome these limitations.

Second, although we classified IFRS based on histopathology, this entity comprises two distinct clinical phenotypes that exhibit heterogeneity in pathophysiology and host immune status: AIFRS (*n* = 34) and CIFRS (*n* = 18). The distinct underlying immune defects of AIFRS versus CIFRS can be characterized as acute, severe immunosuppression or diabetic ketoacidosis in AIFRS versus mild or functional immune deficits in CIFRS. Given the limited sample size of both subgroups and the absence of statistically significant differences in diabetes mellitus, malignant tumors, and other key variables between them, we combined all IFRS cases for multivariate logistic regression analysis to enhance statistical power. We acknowledge this heterogeneity as a limitation. The high OR for diabetes mellitus (5.187) may be predominantly driven by the AIFRS/Mucorales subgroup, particularly those with diabetic ketoacidosis. Thus, the risk profile for an acute fulminant infection differs from that of a chronic invasive process. Risk factors for CIFRS may be more subtle and require separate, adequately powered studies. Future multicenter studies with larger sample sizes should analyze AIFRS and CIFRS separately to delineate their distinct risk profiles.

Third, as a single-center retrospective study conducted at a tertiary otolaryngology center, our investigation may have inherent referral bias (e.g., a higher proportion of severe FRS cases compared to primary care settings). This limits the generalizability of our findings to community or primary care populations in Northern China, and future validation in large-scale multicenter cohorts is needed. Moreover, due to the retrospective design and the fact that some patients were referred from other hospitals, data on key confounders such as immunosuppression (e.g., degree and duration, use of immunomodulatory therapies) and treatment history (e.g., prior antifungal exposure, use of corticosteroids or other immunosuppressants) were potentially incomplete or missing in our institutional records; this lack of completeness may have confounded the associations observed. Additionally, we lacked detailed data on the severity of diabetic ketoacidosis, which may be an important modifier of IFRS risk. Residual confounding from these unmeasured or incompletely captured variables cannot be excluded. Furthermore, the retrospective design precluded a systematic comparative analysis of the efficacy, safety, and prognostic outcomes of different antifungal agents in the management of IFRS, particularly in high-risk subgroups such as patients with diabetes mellitus or mucormycosis.

These unresolved questions highlight several key directions for future research, including prospective multicentre randomised controlled trials to compare the efficacy and safety of different systemic antifungal agents in IFRS patients, especially those with diabetes mellitus or mucormycosis, as well as studies incorporating molecular diagnostic tools (e.g., PCR or metagenomic next-generation sequencing) to provide a more complete pathogen spectrum of FRS in Northern China, so as to optimise the individualised management strategy of IFRS.

In conclusion, this study systematically characterises the clinical and microbiological landscape of FRS in Northern China. The core findings are as follows: (1) FRS in this region is predominantly chronic, with *Aspergillus* spp. (*Aspergillus fumigatus*, *Aspergillus flavus*) as the primary etiological agents; (2) ocular symptoms and nasofacial swelling are highly specific warning signs for IFRS; (3) diabetes mellitus was identified as an independent risk factor associated with IFRS in this cohort. These findings strongly support a stratified, integrated management strategy for FRS, encompassing early and precise diagnosis (combining histopathological and microbiological testing), aggressive surgical debridement, and targeted systemic antifungal therapy. Importantly, for sinusitis patients with diabetes mellitus, the development of ocular or facial symptoms should be recognised as a “red flag” for IFRS, prompting the immediate initiation of a rapid diagnostic pathway involving imaging, microbiological, and histopathological evaluations to improve outcomes in this severe, life-threatening condition.

## Data Availability

Data is provided within the manuscript.

## Abbreviations

FRS: Fungal rhinosinusitis
IFRS: Invasive fungal rhinosinusitis
NIFRS: Non-invasive fungal rhinosinusitis
OR: Odds ratio
CI: Confidence interval
HIS: Hospital information system
LIS: Laboratory information system
CT: Computed tomography
MRI: Magnetic resonance imaging
WBC: White blood cell
NEUT: Neutrophil
EOS: Eosinophil
SDA: Sabouraud dextrose agar
MALDI-TOF MS: Matrix-assisted laser desorption/ionization time-of-flight mass spectrometry
H.E: Haematoxylin and eosin
EPV: Events per variable
FESS: Functional endoscopic sinus surgery
PKC: Protein kinase C
AFRS: Allergic fungal rhinosinusitis
